# From retrospective to prospective design: a short communication on why the methodological paradigm for qualitative research on delayed medical consultation in obstructive sleep apnea may need to be reconstructed

**DOI:** 10.1007/s11325-026-03752-6

**Published:** 2026-06-30

**Authors:** Fei-Yi Zhao, Wen-Jing Zhang, Peijie Xu, Li-Ping Yue, Jie Qian, Qiang-Qiang Fu, Gerard A. Kennedy

**Affiliations:** 1 Department of Nursing, School of International Medical Technology, Shanghai Sanda University, Shanghai, 201209 People’s Republic of China; 2 School of Health and Biomedical Sciences, RMIT University, Bundoora, VIC 3083 Australia; 3 Sydney School of Health Sciences, Faculty of Medicine and Health, The University of Sydney, Camperdown, NSW 2050 Australia; 4 Shanghai Municipal Hospital of Traditional Chinese Medicine, Shanghai University of Traditional Chinese Medicine, Shanghai, 200071 People’s Republic of China; 5 School of Computing Technologies, RMIT University, Melbourne, VIC 3000 Australia; 6 Yangpu Hospital, School of Medicine, Tongji University, Shanghai, 200090 People’s Republic of China; 7 Shanghai Mental Health Center, Shanghai Jiao Tong University School of Medicine, Shanghai, 200030 People’s Republic of China

**Keywords:** Facilitators and barriers, Healthcare-seeking behavior, Healthcare barriers, Seeking medical attention, Patient delay, Treatment delay, Diagnostic delay

## Abstract

**Background and aims:**

Qualitative research offers key insights into enablers and obstacles of healthcare-seeking behavior, and the credibility of findings depends on methodological quality. This study aims to critically appraise the methodological quality of existing qualitative studies on delayed medical consultation in obstructive sleep apnea (OSA) patients, thereby identifying common limitations and offering recommendations for future improvements.

**Methods:**

A systematic search was conducted in EMBASE, MEDLINE, and CENTRAL databases, covering literature from inception to February 2026. Inclusion criteria were qualitative studies (e.g., phenomenological, ethnographic, or grounded theory paradigms) on medical consultation delays in adult OSA patients, excluding single-case studies or narrative reports with very small samples. Two reviewers independently assessed studies against five dimensions, with disagreements resolved by discussion.

**Results:**

Three studies were included, from which eight methodological issues were identified: (1) potential “survivorship bias” from including only diagnosed patients; (2) recall bias in retrospective designs; (3) insufficient consideration of the role of comorbidities in the healthcare-seeking process; (4) unclear directionality between cognitive factors and delayed behavior; (5) lack of in-depth analysis of deviant cases; (6) oversimplified view of family/friend support; (7) insufficient transparency in reporting interview guides and data saturation; and (8) absence of researcher reflexivity during data collection and analysis.

**Conclusions:**

Current qualitative studies on delayed medical consultation in OSA patients have several methodological flaws that may affect their validity. Future research should prioritize prospective, community-based studies targeting high-risk OSA populations not yet seeking medical help, and comprehensively document the entire trajectory of their medical consultation delay. This will enhance research rigor and provide clearer guidance for developing more actionable intervention strategies.

**Supplementary Information:**

The online version contains supplementary material available at 10.1007/s11325-026-03752-6.

## Background

Obstructive sleep apnea (OSA), a prevalent yet underdiagnosed sleep disorder [1], often leads patients to delay care until moderate or severe stages [2]. Cross-sectional surveys have identified multiple factors influencing delayed medical consultation in OSA patients, including perceived barriers to care [3], lower family income [2], reduced self-efficacy [2], and diminished life satisfaction [3]. Qualitative research, which explores the subjective experiences, perceived barriers, and behavioral logic of OSA patients in seeking medical consultation, can complement quantitative studies by offering a more holistic understanding of delays in care-seeking. The methodological rigor of such qualitative research—encompassing study design, implementation, and reporting—directly affects the reliability of its findings [4].

Accordingly, this study appraises the methodology of existing qualitative research on delayed consultation in OSA patients, identifies common weaknesses, and offers recommendations for future studies.

## Methods

### Literature search and screening

A systematic search was conducted in EMBASE (via Ovid), MEDLINE (via PubMed), and CENTRAL to identify qualitative studies on delayed healthcare-seeking behaviors among patients with OSA published up to February 2026. The detailed search strategy and terms are provided in APPENDIX 1.1. To ensure comprehensiveness, reference lists of included studies were also manually screened for additional eligible articles. The search was performed on February 28, 2026, in accordance with the PRISMA-S checklist (APPENDIX 1.2).

Studies were included if they: (1) involved adults diagnosed with OSA; (2) explored delayed medical consultation behaviors, experiences, perceptions, or barriers; (3) employed a qualitative design (e.g., phenomenology, ethnography, or grounded theory); (4) reported original qualitative findings, including those from secondary data analyses; and (5) were published in English.

Studies were excluded if they: (1) were protocols, conference abstracts, or preprints; or (2) were qualitative studies based on single cases or very small samples (e.g., fewer than five participants), such as case studies or narrative reports. These were excluded as they may not adequately capture the heterogeneity of delayed medical consultation behavior in OSA, limiting the transferability of findings.

Records were deduplicated using Zotero (version 7.0). Two reviewers (QQ-F and P-X) independently screened studies according to predefined criteria, with disagreements resolved by a third reviewer (G-K).

### Evaluation dimensions

We evaluated the included qualitative studies across five dimensions: (1) appropriateness of the research paradigm; (2) sampling strategy and sample representativeness; (3) rigor of the data analysis process; (4) researcher reflexivity; (5) completeness and transparency of reporting. Two reviewers (J-Q and LP-Y) independently assessed each study item-by-item. Discrepancies were resolved through team discussions, reaching a consensus.

## Results

Despite a comprehensive search, only three qualitative studies met the eligibility criteria (see APPENDIX 2). During screening, we observed that, compared with the substantial body of qualitative research on illness perceptions, continuous positive airway pressure (CPAP) treatment experience, and treatment adherence, studies explicitly examining delayed healthcare-seeking behavior in OSA are scarce—and the majority of those that do exist are quantitative survey-based designs aimed at identifying contributing factors. This finding suggests that, beyond the identification of “what factors” are associated with delay, the underlying behavioral generation mechanisms—including decision-making processes and behavioral logic—remain largely underexplored at the qualitative level.

Among the three included studies, two were original investigations: one conducted in Israel [1] and the other in China [5], involving face-to-face interviews with 65 Israeli and 15 Chinese OSA patients, respectively. The third was a secondary analysis [6] of a U.S. parent study investigating spousal involvement in CPAP therapy [7]. This analysis focused on 20 couples, including 20 patients with OSA [7]. Methodological appraisal of the included studies identified eight key issues (see Table 1). These issues, along with corresponding recommendations, are elaborated below.

**Table 1:** Study design, analysis, and reporting issues identified across five predefined methodological dimensions

Dimensions of Methodological Assessment	Limitations Identified	Source Article(s)
Sampling Strategy and Sample Representativeness	Potential “Survivorship Bias” due to The Inclusion of Only Diagnosed Patients	①②
Appropriateness of The Research Paradigm	Recall Bias Inherent in Retrospective Designs	①②
Rigor of the Data Analysis Process	Insufficient Consideration of The Role of Comorbidities in The Healthcare-Seeking Process	①②③
	Unclear Directionality between Cognitive Factors and Delayed Behavior	①②③
	Lack of In-Depth Analysis of Deviant Cases	①②
	Oversimplified Understanding of The Role of Family/Friend Support	①②③
Completeness and Transparency of Reporting	Lack of Transparency in Reporting Interview Guides and Data Saturation	①②
Researcher Reflexivity	Absence of Researcher Reflexivity during Data Collection and Analysis	①②③

① Zarhin D [1] Delaying and seeking care for obstructive sleep apnea: The role of gender, family, and morality. Health (London) 22(1): 36–53.

② Shang H, et al. [5] Influencing factors of delay in seeking medical attention of patients with obstructive sleep apnea based on the Model of Pathways to Treatment in China: a qualitative analysis. Sleep Breath 28(5): 2311–2321.

③ Ye L, Li W, and Willis DG [6] Facilitators and barriers to getting obstructive sleep apnea diagnosed: perspectives from patients and their partners. J Clin Sleep Med 18(3): 835–841.

## Discussion

### Avoiding potential “survivorship bias” in sample selection

All three studies included interviewees who had already been diagnosed with OSA. In the two original studies, symptomatic individuals who never sought medical attention or discontinued consultation before diagnosis was confirmed were excluded [1, 5]. Yet these excluded groups constitute the core population of interest in delayed medical consultation. Consequently, these studies are subject to survivorship bias [8], whereby the identified influencing factors primarily reflect experiences of patients who ultimately entered the healthcare system, rather than the broader mechanisms underlying persistent non-consultation. This selection bias may distort the relative importance of influencing factors, overemphasizing individual cognitive and attitudinal determinants while underrepresenting structural barriers, such as limited access to diagnostic equipment/specialists, high transportation costs in remote areas, or inadequate insurance coverage—barriers that may not have been relevant to, or may have already been overcome by, the "survivor" sample.

Further evidence of compromised sample representativeness is seen in the U.S. study, where participants were predominantly male (70%) and middle-aged (mean 49.6 years) [6], and the Chinese study, which also had a predominantly male sample (66.7%) and middle-aged participants (mean 48.9 years) [5]. However, OSA prevalence is also high among postmenopausal women (47%−67%) [9] and older adults (35.1%) [10].

Additionally, the inclusion/exclusion criteria in the Chinese and Israeli studies were insufficiently reported [1, 5], leaving it unclear whether participants were consecutively enrolled or convenience-sampled, or selectively chosen for “stories to tell.”

#### [Recommendation]

Stratified sampling across different stages of healthcare engagement (non-attendance, initial consultation, confirmed diagnosis) is warranted. Specifically, we suggest (1) identifying moderate-to-high-risk individuals from community settings using screening tools (e.g., STOP-Bang questionnaire [11], overnight pulse oximetry [12]), and conducting interviews to explore barriers at the pre-decision stage, and (2) collaborating with sleep centers to track patients who scheduled but did not complete polysomnography, or who completed testing but failed to attend follow-up.

Additionally, maximum variation sampling should be applied to capture diverse demographics [13], along with detailed reporting of recruitment and sampling procedures.

### Navigating recall bias in retrospective designs

Recall bias poses a methodological concern, as the interviewees’ recollections may result in either under- or over-reporting of an event, timelines, experiences, or perceptions, thereby affecting the empirical outcomes and conclusions of the study [14]. In the Israeli study, participants had all received a diagnosis within the preceding 18 months; however, the exact duration of the diagnostic or treatment delay for these patients was not reported [1]. Requiring recall of experiences from up to 18 months prior inherently introduces bias. The researcher also acknowledged that most participants could not provide a precise timeframe, instead offering estimates ranging from several months to multiple years [1]. The recall bias issue is more pronounced in the Chinese study, which employed the Model of Pathways to Treatment (PT-Model) [15], requiring patients to recall four distinct intervals: appraisal (symptom onset to decision to seek care), help-seeking (decision to first consultation), diagnostic (first consultation to definitive diagnosis), and pre-treatment (hesitation before treatment) [5]. Although life event anchoring was utilized in the Chinese study to assist recall, its effectiveness was limited. Patients, particularly those without partners to observe their symptoms, may struggle to accurately identify symptom onset. Moreover, recognizing the need for medical care is a gradual psychological process that is difficult to pinpoint to a specific time point. For 86.7% of participants, the appraisal interval ranged from 5 to 50 years [5], which renders precise recall highly unreliable, compromising the delineation of intervals within the PT-Model and weakening trustworthiness.

#### [Recommendation]

A prospective cohort design is recommended. High-risk populations (e.g., individuals with obesity or habitual snoring) could be recruited from community or health checkup settings, with baseline assessment of symptoms, OSA awareness, and help-seeking intentions, followed by longitudinal tracking of medical consultation behaviors and their triggers. If retrospective designs are unavoidable, multiple data sources should be used to triangulate self-reported timelines [16]—for example, interviewing bed partners to corroborate symptom onset, and consulting primary care or insurance records to identify earlier symptom-related consultations.

### Distinguishing active help-seekers from passive discoverers based on the consideration of comorbidities

OSA patients frequently have comorbidities [17], which can also influence medical consultation. However, the three studies reviewed devoted limited attention to the complex relationship between comorbidities and delayed consultation in OSA patients [1, 5, 6].

First, among OSA patients with comorbidities such as cardiovascular and metabolic diseases, a substantial proportion may be “passive discoverers” rather than “active help-seekers.” Active help-seekers initiate medical consultation in response to perceived OSA-related symptoms (e.g., snoring and excessive daytime sleepiness), and their delay is shaped largely by symptom recognition and decision-making processes, with symptom attribution, perceived severity, and health beliefs potentially acting as key determinants. By contrast, passive discoverers are incidentally diagnosed during healthcare encounters for other conditions (e.g., hypertension or diabetes). Their healthcare use is driven by comorbidities, with OSA as an unintentional finding. In this group, delay may arise from asymptomatic presentation or the masking of OSA symptoms by more salient comorbid conditions. None of the three studies distinguished between these subgroups, instead analyzing them collectively, which may limit the value of the findings for guiding targeted intervention development. By contrast, adequate consideration of comorbidities and stratification of participants might better enhance the interpretability of findings and the reference value of the results. For instance, strategies to reduce delayed consultation may need to integrate public awareness initiatives targeting active help-seekers, with opportunistic OSA screening within chronic disease management programs particularly relevant for passive discoverers.

Second, the role of comorbidities in delayed consultation should not be interpreted in a unidirectional manner as either facilitative or hindering. Under multimorbidity, patients may attribute OSA-related symptoms (e.g., daytime fatigue, difficulty concentrating [18]) to more familiar conditions (e.g., hypertension, stress, or aging), thereby prolonging the appraisal interval. However, the Chinese study primarily framed comorbidities as facilitators of healthcare contact (e.g., consulting for hypertension and incidentally identifying OSA), overlooking their confounding and masking roles [5], which results in an incomplete account of delay mechanisms.

#### [Recommendation]

Multimorbidity should be treated as a central analytic dimension rather than a background variable. Frameworks such as illness trajectory [19] or symptom network models [20] may be applied to examine temporal sequencing, interrelations among conditions, and their joint effects on medical consultation behavior, thereby more accurately revealing how OSA is perceived, prioritized/deprioritized, and acted upon in the context of comorbidities.

### Further exploring the directionality between cognition and delay

Studies from both the U.S. and Israel attribute delays in medical care to patients’ adaptation to symptoms such as fatigue and snoring [1, 6]. Similarly, research in China identifies insufficient awareness of OSA and its associated risks—such as not interpreting snoring as a warning sign, or even viewing it as a blessing—as a primary driver of delayed medical consultation [5]. While plausible, this interpretation may reflect circular reasoning. Prolonged delay without overt adverse consequences may foster cognitive rationalization of inaction, consistent with cognitive dissonance reduction theory [21]: when a behavior (e.g., ignoring snoring or not seeking care) persists without catastrophic outcomes, individuals may develop beliefs that justify it (e.g., “nothing has happened, so it is not a serious disease”). As Participant No. 1 in the Chinese study stated: “...*we all snored… so you can’t say they were all sick*” [5]. Similarly, Michael in the Israeli study remarked, “*If something hurts*,* I let it go—it will go away on its own*,” often viewing fatigue, sleepiness, and snoring as inevitable parts of daily life: “*we are struggling to balance personal and professional life*,* and to meet multiple social obligations*,* including work and family*,* … so fatigue and sleepiness are ‘unsurprising’*” [1].

Relying on retrospective interviews to capture participants’ current beliefs and then infer causal relationships may not be entirely reasonable, as such designs cannot readily differentiate whether these beliefs preceded the delay or emerged as post-hoc rationalizations. Conflating the two may result in an inflated expectation of intervention efficacy – for instance, the belief that a unifocal educational intervention focusing solely on awareness improvement can effectively reduce delay. But in reality, for individuals with entrenched rationalization beliefs, simple educational interventions may be insufficient, and cognitive restructuring may be required.

#### [Recommendation]

Distinguishing beliefs at symptom onset from those formed during prolonged delay is important. Interviews may incorporate temporal probing (e.g., “*What was your initial reaction when you first noticed or were told about your snoring?*” and “*How have your views changed over time*,* and what influenced these changes?*”).

A better approach would be adopting longitudinal designs—prospective follow-up of high-risk populations with repeated assessments of health beliefs, risk perception, and help-seeking intentions can elucidate the dynamic relationship between cognition and behavior and clarify whether beliefs function as antecedents or consequences of delayed OSA medical consultation.

### Incorporating divergent cases to enrich qualitative analysis

Although all three studies identified insufficient disease awareness as a key factor contributing to delayed medical consultation for OSA, some participants exhibited relatively high levels of awareness. For instance, in the Chinese study, Participant No. 5 noted that “*OSA can cause cardiovascular diseases*” and that she had “*looked into it before*,” while Participant No.2 linked weight gain to OSA and understood snoring as a symptom of the disease [5]. Similarly, in the Israeli study, Participant Jacob reported undergoing sleep tests twice—once in the U.S. in 2003 and again later in Israel—yet his awareness did not alter his delayed consultation behavior [1]. These cases, contrary to the study’s predominant narrative of low awareness, were not systematically explored.

Why did these participants, despite greater awareness, still experience delays? Deeper psychological mechanisms may be at play, such as rejection of the disease label, a luck-oriented mindset (“*Nothing will happen if I don’t treat it*”), or latent resistance to CPAP, oral appliances, or surgery. This hypothesis is supported by findings from the U.S. study, which also identified high-awareness patients and examined the underlying reasons for their delayed consultation. The study concluded that illness denial, the inconvenience of sleep testing, anticipated high costs, and the cumbersome nature of CPAP treatment contributed to delayed consultations [6]. One participant recalled: “*I was in denial about it (OSA)…I didn’t want to go through that process of sleep study…*”; others expressed reluctance toward “*wrapping like a lie detector …with all those wires and the lack of privacy*.” Another participant commented, “*I have heard horror stories that CPAP is an extreme cost.so I think a lot of potential patients put all these barriers in their head*” [6].

Had the Chinese and Israeli studies also analyzed these deviant cases, they might have offered a more nuanced understanding of delayed consultation.

#### [Recommendation]

The identification and interpretation of deviant cases could be treated as a core analytic task. This may involve purposive inclusion of extreme or contrasting cases and their systematic examination as independent themes or subthemes using deviant case analysis strategies [22], thereby uncovering less visible but critical determinants.

In addition, given the study focuses on influencing factors, attention should also be directed to individuals who sought care promptly. Exploring facilitators—such as strong health awareness, heightened risk perception, professional guidance from medically trained family members, or salient triggering events (e.g., publicized deaths related to OSA complications)—may offer a complementary perspective to provide actionable targets for intervention design.

### Developing a more nuanced understanding of the role of family/friend support

Both Chinese and U.S. studies propose that social support from relatives, friends and/or spouses may shorten delays in healthcare-seeking [5, 6]. The Israeli study, however, presents two contrasting perspectives. It reports findings consistent with the Chinese and U.S. studies, where reminders or complaints from partners prompt medical consultations, yet it also highlights instances such as participant Jacob’s, where, despite acknowledging his wife’s influence, he emphasized that her complaints were not the primary motivation for his decision to seek medical help [1]. These findings align with prior evidence that early disclosure of symptoms to significant others (e.g., family, friends, and neighbours) may support timely healthcare utilization, though the association is not consistently observed [15].

Notwithstanding these insights, the potentially adverse effects of family/friend support appear underexplored in all three studies. First, family members may engage in symptom minimization or concealment, intentionally or unintentionally, to preserve the patient’s self-esteem and avoid conflict. Second, when snoring is prevalent within the family, it may become normalized within the social context, leading to a shared belief that it is not indicative of disease. In this context, family support may not facilitate medical consultation but instead reinforce misconceptions that hinder it. One participant in the U.S. study delayed seeking medical attention, stating, “*my older son has his own health issues*…,” implying that some health issues are common and do not warrant medical intervention [6]. Similarly, a participant in the Chinese study recalled: “*I used to live in the army dormitory*,* and found that we all snored… so you can’t say they were all sick*” [5].

#### [Recommendation]

Dyadic designs are recommended, interviewing patients alongside cohabiting family members (particularly spouses). Examining interaction patterns, communication processes, and the co-construction of illness perceptions of OSA may reveal relational dynamics and tensions not captured in individual interviews. Furthermore, analyses should draw on established social support frameworks to distinguish informational, emotional, and instrumental support, and examine their differential effects on healthcare-seeking decisions and delays.

### Ensuring greater transparency in interview guide and theoretical saturation procedures

In both the Israeli and Chinese studies, although the authors claimed to have developed interview guides, neither reported their content nor the principles underlying their design [1, 5]. This omission precludes assessing the neutrality, directionality, and comprehensiveness of the interview questions in capturing factors influencing delayed consultation. It also remains unclear whether the questions allowed free expression or merely validated pre-existing frameworks.

Additionally, both studies inadequately described the process for determining theoretical saturation. The Chinese study only stated that interviews ceased when “no new themes” emerged [5]. The Israeli study referenced criteria from other research, stating that saturation was reached when data provided detailed descriptions, category properties were developed, and further interviews did not yield new insights into the emerging concepts [1]. However, neither study outlined the analytic process underlying this judgment, including coding framework development, theme derivation and refinement, or the point at which thematic redundancy was reached [1, 5]. Thus, it is difficult to ascertain whether saturation was genuinely achieved. Importantly, theoretical saturation entails not only the absence of new themes but also the full elaboration of existing categories and their properties.

#### [Recommendation]

Future studies should include the complete interview guide, along with a brief account of its design principles, to enhance transparency and facilitate appraisal, comparison, and replication. Researchers should clarify how openness was ensured and how leading questions were avoided.

Regarding data saturation, structured approaches such as the “Principles for Specifying Data Saturation” proposed by Francis et al. are recommended [23]. This involves defining an initial analysis sample, followed by iterative data collection in pre-specified batches until predefined stopping criteria are met. Such procedures transform saturation determination from a subjective assertion into a verifiable research procedure, thereby enhancing the credibility and reproducibility of qualitative findings [24].

### Integrating researcher reflexivity to strengthen analytical rigor

The researcher’s background, professional experience, cultural positioning, and potential biases inevitably shape qualitative data collection and interpretation [25]. Reflexivity is therefore essential to ensure rigor, transparency, and credibility [26, 27]. In the U.S. study, both interviewers were advanced practice registered nurses [6]. In the Chinese and Israeli studies, the researchers did not report whether the interviewers were clinicians, nurses, or independent research assistants [1, 5]. If interviewers were medical professionals, the inherent power asymmetry in clinician–patient relationships may have predisposed participants to provide socially desirable responses, to align with perceived medical expectations, or to withhold critical views of the healthcare system, thereby compromising data authenticity.

Reflexive consideration of analytical bias is also lacking. It is unclear how the authors mitigated the risk of imposing theoretical assumptions (e.g., the PT-Model) onto the data—such as selectively privileging confirmatory evidence while overlooking discrepant findings—and how these assumptions influenced coding and theme development. This absence limits readers’ ability to evaluate the study’s credibility. More critically, in the Israeli study, both the interviews and data analysis were conducted by a single researcher—the author [1]. Without triangulation [16], no explanation is provided regarding how bias was managed or how coding reliability was ensured [1].

#### [Recommendation]

The professional background of interviewers and analysts, as well as their relationship to the research topic, should be explicitly reported. To minimize power-related bias, interviews may be conducted by clinical staff without a direct therapeutic relationship with participants, or preferably by trained non-clinical qualitative researchers.

Maintaining reflexive journals throughout data collection and analysis is also advisable, documenting impressions, assumptions, and potential biases [26, 27]. Selected excerpts may be reported to demonstrate how reflexivity informed the analytic process, thereby enhancing transparency and trustworthiness.

## Conclusions

Our current findings support advocating for a paradigm shift from retrospective sampling of already-diagnosed patients to a prospective, community-anchored design targeting high-risk individuals who have not yet sought care. This approach would better capture the complete trajectory of delayed healthcare-seeking behavior in OSA patients.

Enhancing methodological rigor and sophistication in qualitative research can improve the comprehensiveness and credibility of findings, thereby generating actionable insights—including guidance for developing (1) health education interventions targeting key warning symptoms (e.g., habitual snoring and excessive daytime sleepiness) to reshape patients’ symptom attribution patterns; (2) partner- and family-centered interventions to strengthen patients’ social support and reduce disease normalization; (3) community-based targeted screening strategies to identify at-risk individuals not yet seeking medical consultation; (4) standardized integration of OSA screening into routine chronic disease management; (5) training programs for primary care providers to improve their OSA awareness, recognition, and proactive referral practices; and (6) streamlined referral pathways to minimize delays and loss to follow-up between initial screening and diagnostic confirmation. Collectively, these efforts may ultimately dismantle both individual-level barriers (e.g., psychosocial factors such as denial or low perceived risk of the disease) and structural barriers (e.g., inadequate screening and diagnostic capacity in primary care, and inefficient community-specialist referral pathways) that impede timely diagnosis and treatment of OSA—a critical step toward alleviating the substantial public health burden imposed by healthcare-seeking delay.

## Supplementary Information

Below is the link to the electronic supplementary material.

Supplementary Material 1 (PDF 628 KB)

## Data Availability

My manuscript has no associated data.

## Abbreviations

CPAP: Continuous Positive Airway Pressure
OSA: Obstructive Sleep Apnea
PT-Model: Model of Pathways to Treatment
