# Supplementary material for: From retrospective to prospective design: a short communication on why the methodological paradigm for qualitative research on delayed medical consultation in obstructive sleep apnea may need to be reconstructed
Source: Sleep Breath. 2026 Jun 30;30(4):205. doi: 10.1007/s11325-026-03752-6 (PMC13319258; doi:10.1007/s11325-026-03752-6)
Supplement: Supplementary file 1 — Supplementary Material 1 (PDF 628 KB) [file 11325_2026_3752_MOESM1_ESM.pdf]

# *Supplementary Materials*

**From Retrospective to Prospective Design: A Short Communication on Why the Methodological Paradigm for Qualitative Research on Delayed Medical Consultation in Obstructive Sleep Apnea May Need to Be Reconstructed**

Fei-Yi Zhao[1,2,3,4†], Wen-Jing Zhang[4†], Peijie Xu[5†], Li-Ping Yue[1†], Jie Qian[6,7*], Qiang-Qiang Fu[6*], Gerard A. Kennedy[2]

[1] *Department of Nursing, School of International Medical Technology, Shanghai Sanda University, Shanghai, 201209, People's Republic of China*

[2] *School of Health and Biomedical Sciences, RMIT University, Bundoora, VIC, 3083, Australia*

[3] *Sydney School of Health Sciences, Faculty of Medicine and Health, The University of Sydney, Camperdown, NSW, 2050, Australia*

[4] *Shanghai Municipal Hospital of Traditional Chinese Medicine, Shanghai University of Traditional Chinese Medicine, Shanghai, 200071, People's Republic of China*

[5] *School of Computing Technologies, RMIT University, Melbourne, VIC, 3000, Australia*

[6] *Yangpu Hospital, School of Medicine, Tongji University, Shanghai, 200090, People's Republic of China*

[7] *Shanghai Mental Health Center, Shanghai Jiao Tong University School of Medicine, Shanghai, 200030, People's Republic of China*

† These authors contributed equally to this work

* ***Correspondence:***

Qiang-Qiang Fu, Yangpu Hospital, School of Medicine, Tongji University, Shanghai, 200090, People's Republic of China, Tel: (+ 86) 021-6569 0520, Fax: (+ 86) 021-6569 6249; Email: qiangqiang.fu@tongji.edu.cn

Jie Qian, Yangpu Hospital, School of Medicine, Tongji University, Shanghai, 200090, People's Republic of China, Tel: (+ 86) 021-6569 0520, Fax: (+ 86) 021-6569 6249; Email: bsb.qianjie@tongji.edu.cn

**APPENDIX 1 Search Strategy for a Qualitative Study on Delayed Medical Consultation in OSA Patients**

**APPENDIX 1.1 Search Strategy for Comprehensive English Databases**

| Databases | Search Strategy |
|---|---|
| MEDLINE (via PubMed) | #1 "Sleep Apnea, Obstructive"[MeSH Terms]<br>#2 "Sleep Apnea, Obstructive"[Title/Abstract]<br>#3 "OSA"[Title/Abstract]<br>#4 "OSAHS"[Title/Abstract]<br>#5 "Sleep-disordered breathing"[Title/Abstract]<br>#6 "sleep apnea syndrome"[Title/Abstract]<br>#7 "Obstructive Sleep Apnea"[Title/Abstract]<br>#8 "sleep apnea"[Title/Abstract]<br>#9 "obstructive sleep apnoea"[Title/Abstract]<br>#10 "obstructive sleep apnea hypopnea syndrome"[Title/Abstract]<br>#11 #1 OR #2 OR #3 OR #4 OR #5 OR #6 OR #7 OR #8 OR #9 OR #10<br>#12 "treatment delay"[Title/Abstract]<br>#13 "Patient Delay"[Title/Abstract]<br>#14 "Diagnostic Delay"[Title/Abstract]<br>#15 "Health-seeking delay"[Title/Abstract]<br>#16 "healthcare delay"[Title/Abstract]<br>#17 "medical care delay"[Title/Abstract]<br>#18 "help-seeking delay"[Title/Abstract]<br>#19 "Delay in Seeking Care"[Title/Abstract]<br>#20 "Delay in Seeking Treatment"[Title/Abstract]<br>#21 "barriers to care"[Title/Abstract]<br>#22 "care delay"[Title/Abstract]<br>#23 "access to care barriers"[Title/Abstract]<br>#24 "treatment-seeking delay"[Title/Abstract]<br>#25 "diagnosis delay"[Title/Abstract]<br>#26 #12 OR #13 OR #14 OR #15 OR #16 OR #17 OR #18 OR #19 OR #20 OR #21 OR #22 OR #23 OR #24 OR #25<br>#27 "qualitative research"[MeSH Terms]<br>#28 "qualitative research"[Title/Abstract]<br>#29 "qualitative study"[Title/Abstract]<br>#30 "phenomenology"[Title/Abstract]<br>#31 "grounded theory"[Title/Abstract]<br>#32 "ethnography"[Title/Abstract]<br>#33 "narrative inquiry"[Title/Abstract]<br>#34 "thematic analysis"[Title/Abstract]<br>#35 "content analysis"[Title/Abstract]<br>#36 "focus groups"[MeSH Terms]<br>#37 "focus groups"[Title/Abstract]<br>#38 "interview"[Title/Abstract]<br>#39 "Interviews as Topic"[MeSH Terms] |

| | |
|---|---|
| | #40 qualitative analys*[Title/Abstract]<br>#41 field study[Title/Abstract]<br>#42 ethnologic*[Title/Abstract]<br>#43 #27 OR #28 OR #29 OR #30 OR #31 OR #32 OR #33 OR #34 OR #35 OR #36 OR #37 OR #38 OR #39 OR #40 OR #41 OR #42<br>#44 #11 AND #26 AND #43 |
| Cochrane Central Register of Controlled Trials (CENTRAL) | #1 MeSH descriptor: [Sleep Apnea, Obstructive] explode all trees<br>#2 "Sleep Apnea, Obstructive":ti,ab,kw<br>#3 OSA:ti,ab,kw<br>#4 OSAHS:ti,ab,kw<br>#5 "Sleep-disordered breathing":ti,ab,kw<br>#6 "sleep apnea syndrome":ti,ab,kw<br>#7 "Obstructive Sleep Apnea":ti,ab,kw<br>#8 "sleep apnea":ti,ab,kw<br>#9 "obstructive sleep apnoea":ti,ab,kw<br>#10 "obstructive sleep apnea hypopnea syndrome":ti,ab,kw<br>#11 #1 OR #2 OR #3 OR #4 OR #5 OR #6 OR #7 OR #8 OR #9 OR #10<br>#12 "treatment delay":ti,ab,kw<br>#13 "Patient Delay":ti,ab,kw<br>#14 "Diagnostic Delay":ti,ab,kw<br>#15 "Health-seeking delay":ti,ab,kw<br>#16 "healthcare delay":ti,ab,kw<br>#17 "medical care delay":ti,ab,kw<br>#18 "help-seeking delay":ti,ab,kw<br>#19 "Delay in Seeking Care":ti,ab,kw<br>#20 "Delay in Seeking Treatment":ti,ab,kw<br>#21 "barriers to care":ti,ab,kw<br>#22 "care delay":ti,ab,kw<br>#23 "access to care barriers":ti,ab,kw<br>#24 "treatment-seeking delay":ti,ab,kw<br>#25 "diagnosis delay":ti,ab,kw<br>#26 #12 OR #13 OR #14 OR #15 OR #16 OR #17 OR #18 OR #19 OR #20 OR #21 OR #22 OR #23 OR #24 OR #25<br>#27 MeSH descriptor: [Qualitative Research] explode all trees<br>#28 "qualitative research":ti,ab,kw<br>#29 qualitative stud*:ti,ab,kw<br>#30 phenomenolog*:ti,ab,kw<br>#31 grounded theor*:ti,ab,kw<br>#32 ethnograph*:ti,ab,kw<br>#33 "narrative inquiry":ti,ab,kw<br>#34 "thematic analysis":ti,ab,kw<br>#35 "content analysis":ti,ab,kw<br>#36 MeSH descriptor: [Focus Groups] explode all trees<br>#37 focus group*:ti,ab,kw<br>#38 interview*:ti,ab,kw |

| | |
|---|---|
| | #39 MeSH descriptor: [Interviews as Topic] explode all trees<br>#40 qualitative analys*:ti,ab,kw<br>#41 field stud*:ti,ab,kw<br>#42 ethnologic*:ti,ab,kw<br>#43 #27 OR #28 OR #29 OR #30 OR #31 OR #32 OR #33 OR #34 OR #35 OR #36 OR #37 OR #38 OR #39 OR #40 OR #41 OR #42<br>#44 #11 AND #26 AND #43 |
| EMBASE (via Ovid) | #1 obstructive sleep apnea/exp<br>#2 "Sleep Apnea, Obstructive".ti,ab.<br>#3 OSA.ti,ab.<br>#4 OSAHS.ti,ab.<br>#5 "Sleep-disordered breathing".ti,ab.<br>#6 "sleep apnea syndrome".ti,ab.<br>#7 "Obstructive Sleep Apnea".ti,ab.<br>#8 "sleep apnea".ti,ab.<br>#9 "obstructive sleep apnoea".ti,ab.<br>#10 "obstructive sleep apnea hypopnea syndrome".ti,ab.<br>#11 #1 OR #2 OR #3 OR #4 OR #5 OR #6 OR #7 OR #8 OR #9 OR #10<br>#12 "treatment delay".ti,ab.<br>#13 "Patient Delay".ti,ab.<br>#14 "Diagnostic Delay".ti,ab.<br>#15 "Health-seeking delay".ti,ab.<br>#16 "healthcare delay".ti,ab.<br>#17 "medical care delay".ti,ab.<br>#18 "help-seeking delay".ti,ab.<br>#19 "Delay in Seeking Care".ti,ab.<br>#20 "Delay in Seeking Treatment".ti,ab.<br>#21 "barriers to care".ti,ab.<br>#22 "care delay".ti,ab.<br>#23 "access to care barriers".ti,ab.<br>#24 "treatment-seeking delay".ti,ab.<br>#25 "diagnosis delay".ti,ab.<br>#26 #12 OR #13 OR #14 OR #15 OR #16 OR #17 OR #18 OR #19 OR #20 OR #21 OR #22 OR #23 OR #24 OR #25<br>#27 "qualitative research".ti,ab.<br>#28 "qualitative research".ti,ab.<br>#29 qualitative stud$.ti,ab.<br>#30 phenomenolog$.ti,ab.<br>#31 grounded theor$.ti,ab.<br>#32 ethnograph$.ti,ab.<br>#33 "narrative inquiry".ti,ab.<br>#34 "thematic analysis".ti,ab.<br>#35 "content analysis".ti,ab.<br>#36 focus group$.ti,ab.<br>#37 focus group$.ti,ab. |

| | |
|---|---|
| | #38 interview$.ti,ab.<br>#39 interview$.ti,ab.<br>#40 qualitative analys$.ti,ab.<br>#41 field stud$.ti,ab.<br>#42 ethnologic$.ti,ab.<br>#43 #27 OR #28 OR #29 OR #30 OR #31 OR #32 OR #33 OR #34 OR #35 OR #36 OR #37 OR #38 OR #39 OR #40 OR #41 OR #42<br>#44 #11 AND #26 AND #43 |

**APPENDIX 1.2 The literature Search in This Study is Conducted in accordance with the PRISMA-S Checklist**

| Section/topic | # | Checklist item | Location(s) Reported |
|---|---|---|---|
| **INFORMATION SOURCES AND METHODS** | | | |
| Database name | 1 | Name each individual database searched, stating the platform for each. | 2.1 Literature Search and Screening |
| Multi-database searching | 2 | If databases were searched simultaneously on a single platform, state the name of the platform, listing all of the databases searched. | N/A |
| Study registries | 3 | List any study registries searched. | N/A |
| Online resources and browsing | 4 | Describe any online or print source purposefully searched or browsed (e.g., tables of contents, print conference proceedings, web sites), and how this was done. | N/A |
| Citation searching | 5 | Indicate whether cited references or citing references were examined, and describe any methods used for locating cited/citing references (e.g., browsing reference lists, using a citation index, setting up email alerts for references citing included studies). | 2.1 Literature Search and Screening |
| Contacts | 6 | Indicate whether additional studies or data were sought by contacting authors, experts, manufacturers, or others. | 2.1 Literature Search and Screening |
| Other methods | 7 | Describe any additional information sources or search methods used. | Reference Lists of Included Studies (2.1 Literature Search and Screening) |
| **SEARCH STRATEGIES** | | | |
| Full search strategies | 8 | Include the search strategies for each database and information source, copied and pasted exactly as run. | Appendix 1 of the Supplementary Material |
| Limits and restrictions | 9 | Specify that no limits were used, or describe any limits or restrictions applied to a search (e.g., date or time period, language, study design) and provide justification for their use. | 2.1 Literature Search and Screening |

| | | | |
|---|---|---|---|
| Search filters | 10 | Indicate whether published search filters were used (as originally designed or modified), and if so, cite the filter(s) used. | 2.1 Literature Search and Screening |
| Prior work | 11 | Indicate when search strategies from other literature reviews were adapted or reused for a substantive part or all of the search, citing the previous review(s). | N/A |
| Updates | 12 | Report the methods used to update the search(es) (e.g., rerunning searches, email alerts). | N/A |
| Dates of searches | 13 | For each search strategy, provide the date when the last search occurred. | 2.1 Literature Search and Screening |
| **PEER REVIEW** | | | |
| Peer review | 14 | Describe any search peer review process. | Dual-Person Retrieval and Screening (2.1 Literature Search and Screening) |
| **MANAGING RECORDS** | | | |
| Total Records | 15 | Document the total number of records identified from each database and other information sources. | Appendix 2 of the Supplementary Material |
| Deduplication | 16 | Describe the processes and any software used to deduplicate records from multiple database searches and other information sources. | Zotero, version 7.0 (2.1 Literature Search and Screening) |

***Notes:*** PRISMA-S Checklist was adapted from ***Rethlefsen ML, Kirtley S, Waffenschmidt S, Ayala AP, Moher D, Page MJ, Koffel JB; PRISMA-S Group. PRISMA-S: an extension to the PRISMA statement for reporting literature searches in systematic reviews. J Med Libr Assoc. 2021 Apr 1;109(2):174-200. doi: 10.5195/jmla.2021.962***. Creative Commons

**APPENDIX 2 Flow Diagram of the Literature Selection**

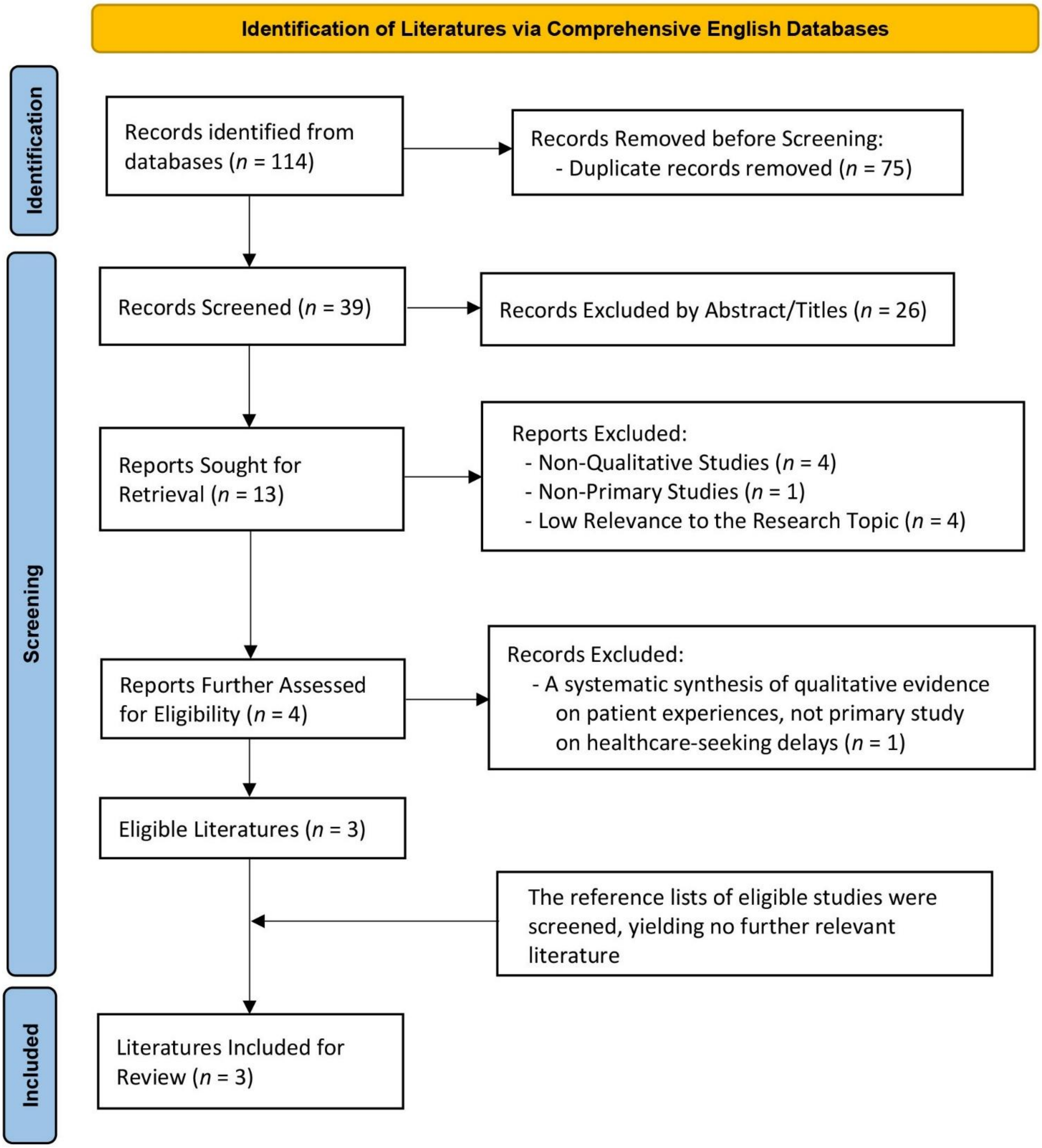


***Notes:*** PRISMA Figure was adapted from **Page MJ, McKenzie JE, Bossuyt PM, *et al*. The PRISMA 2020 statement: an updated guideline for reporting systematic reviews. *BMJ*, 2021; 372: n71 doi:10.1136/bmj.n71.** Creative Commons
